# Case of a sigmoid colon cancer with metachronous metastases to the mesorectum and the abdominal wall

**DOI:** 10.1186/1477-7819-8-17

**Published:** 2010-03-21

**Authors:** John Kalaitzis, George Filippou, Adamantia Zizi-Sermpetzoglou, Athanasios Marinis, Andreas Hadjimarcou, Nikolaos Paschalidis, Spyros Rizos

**Affiliations:** 1 1stDepartment of Surgery, General Hospital of Pireus Tzaneio, Athens, Greece

## Abstract

**Backround:**

Sigmoid colon cancer metachronous metastases commonly occur in the liver and lungs with sporadic reports also to the spleen, stomach, thyroid gland, abdominal wall and upper urinary tract. This is a rare case of metachronous metastases invading the mesorectum and the abdominal wall.

**Case presentation:**

A 72-year-old female underwent sigmoidectomy for stage I (T2N0 M0) sigmoid colon cancer in May 2008. In June 2009, an abdominal computed tomography scan revealed a tumor 2 cm in size at the lower anterior mesorectum and a second mass 2 cm in size at the anterior abdominal wall midline. Total colonoscopy showed no mucosal lesion. The serum carcinoembryonic antigen level was normal. A biopsy of the mesorectum tumor showed similar histologic characteristics with the primary tumor. Since no other site of recurrence was identified, an abdominoperineal resection was attempted. During the operation and after the removal of the incision recurrence, sinus bradycardia and signs of myocardial ischemia were noticed. A loop transverse colostomy was immediately perfomed and the operation was terminated. Postoperative cardiologic examination revealed an acute myocardium infract. Chemo-radiation of the mesorectum tumor and re-evaluation for surgical excision was decided.

**Conclusion:**

Metachronous metastasis of the mesorectum from sigmoid colon cancer is extremely rare. Although patterns of lymphatic spread from rectal cancer to sigmoid colon have recently been demonstrated, there is no evidence of metachronous mesorectum invasion from sigmoid colon cancer. This could be the issue for future trials.

## Background

Colon cancer as one of the most commonly diagnosed cancers around the world has an improved prognosis due to the development of diagnostic and therapeutic procedures. Overall survival however, can be seriously shortened, mostly in the presence of distant metastasis during follow-up. Liver is the commonest site of metachronous metastases in approximately one fourth of the patients, followed by the lungs [1]. Sporadic reports also demonstrate the spleen, thyroid gland, stomach, urinary system and abdominal wall as sites of possible reccurence [2-4]. This is a rare case of a sigmoid colon cancer with metachronous metastases of the mesorectum and the abdominal wall.

## Case Presentation

**Figure 1:**
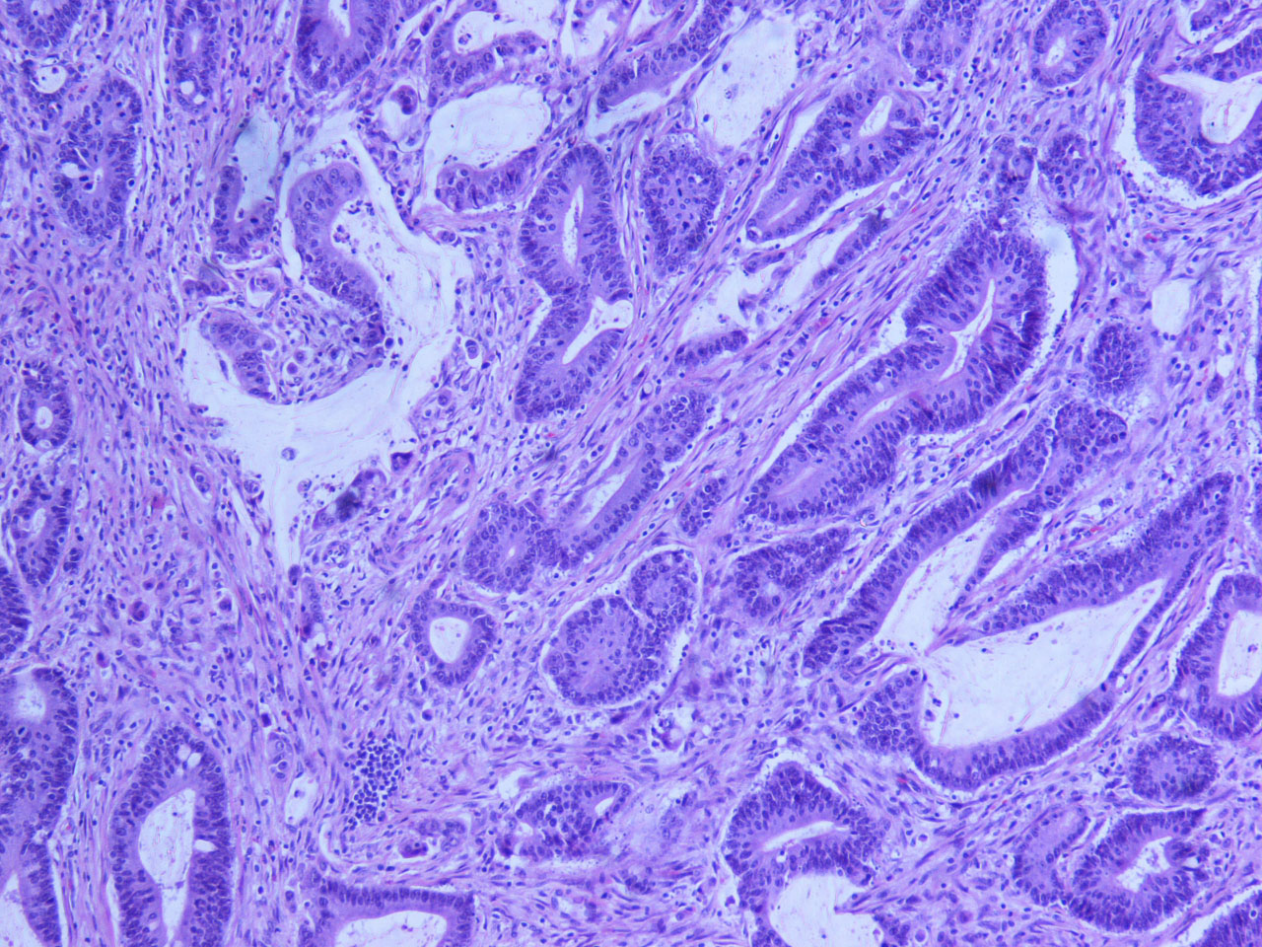
**Moderately differentiated adenocarcinoma (HE × 200)**. The glands are lined by neoplastic cells with hyperchromatic nuclei. The tumor was accompanied with small mucinous lakes lind by well-differentiated epithelial cells. The tumor invades all intestinal wall.

**Figure 2:**
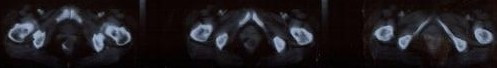
**A pelvic CT showed a tumor 2 cm in size at the lower anterior mesorectum**.

**Figure 3:**
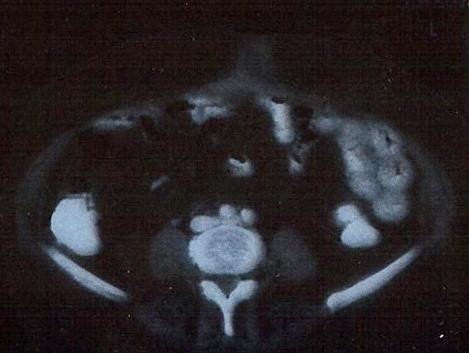
**an Abdominal CT showed enhanced wall thickening in the anterior wall midline**.

A 72-year-old female was admitted to hospital suffering from fatigue, weight loss and rectal bleeding. Total colonoscopy demonstrated adenocarcinoma of the sigmoid colon at 25 cm from the anal verge. A chest x-ray was normal. No sites of distant metastasis were reported on abdominal computed tomography (CT) scan. The serum carcinoembryonic antigen level was normal. The patient underwent sigmoidectomy in May 2008. During the operation, the left ovary was fixed at the site of the sigmoid colon cancer and was removed en block. Thorough macroscopic examination of the liver and rest of the abdomen showed no sign of metastatic disease. Histopathological examination of the specimen revealed a moderately differentiated mucus-producing adenocarcinoma, 3 cm in diameter located 5 cm from the peripheral surgical margin (figure 1). The tumor invaded into but not beyond the muscularis propria (T2). Ki 67 antigen and p53 tumor suppressor protein staining were positive and epidermal growth factor receptor (EGFR) negative. The left ovary was free of neoplasmatic tissue. Only four lymph nodes were counted, free of metastatic adenocarcinoma. The patient had an uneventful recovery. On rectum examination one year later a palpable extramucosal mass was noticed at the anterior rectum wall. An abdominal CT scan revealed a tumor 2 cm in size at the lower anterior mesorectum in close relation with the posterior vaginal wall and a second mass 2 cm in size at the anterior abdominal wall midline (figure 2, 3). Total colonoscopy showed no mucosal lesion. A chest x-ray was normal. Rectal endoscopic ultrasound (EUS) showed a tumor infiltrating the rectum muscularis propria from outside. Core needle biopsy demonstrated the presence of a mucus producing adenocarcinoma with the same histological futures with the primary tumor and therefore it was considered as metachronous metastasis. Serum carcinoembryonic antigen level was normal. Since no other site of recurrence was identified, an abdominoperineal resection was attempted [5]. At laparotomy, the anterior abdominal wall mass was located at the site of previous incision and after complete resection, fast biopsy showed adenocarcinoma. Surgical examination of the abdominal cavity showed no sign of reccurence. At that time, sinus bradycardia and ST segment depression was noticed on electrocardiogram (ECG) monitoring. The termination of the operation was decided and a loop transverse colostomy was immediately perfomed. Postoperative cardiologic examination revealed an acute muocardium infract and the patient was treated respectively. Chemo-radiation of the mesorectum tumor and re-evaluation for surgical excision was decided and she was discharged on the eleventh post operative day.

## Conclusion

Recurrence after curative resection of colon adenocarcinoma is a major determinant of survival and it seems to increase as the primary tumor stage advance. In this case, a sigmoid adenocarcinoma is infiltrating 2/3 of muscularis propria with four lymph nodes harvested free of metastatic disease. According to TNM classification this is a stage I (TIN0 M0) colon cancer but with inadequate lymph node evaluation [6-8]. Even though the literature lacks consensus as to what the exact number of lymph nodes by stage should be, a minimal number of twelve lymph nodes is recommended [9-13]. Nevertheless, metachronous metastasis to the mesorectum of a moderately differentiated T2 sigmoid adenocarcinoma with adequate peripheral surgical margin is extremely rare. The pathway tumor cells followed is unknown but one could suspect lymphatic spread. This is justified by the inadequate lymph node excision. But even if this is true, downward lymphatic flow is not common [14,15]. Recently, lymphatic spread from rectal cancer to the sigmoid colon lymph nodes was demonstrated in as much as 20% [16]. It seems that lymph flow is not only towards the paraortic lymph nodes but follows a more diffuse pattern. In addition, it is known that wound recurrence incidence is less than 1% and is probably a result of implanted cancer cells on the surgical wound during removal of the surgical specimen [17].

This case is important in showing that metachronous metastasis of the mesorectum from sigmoid colon cancer is possible, probably due to downward lymphatic cancer spread. Perhaps, there is a need of a more thorough investigation of the mesorectum region in sigmoid colon cancer during preoperative imaging. There is no similar report published in the literature.

## Consent

Written informed consent was obtained from the patient for publication of this case report and any accompanying images. A copy of the written consent is available for review by the Editor-in-Chief of this journal.

## Competing interests

The authors declare that they have no competing interests.

## Authors' contributions

JK first noticed this rare case report and wrote the main manuscript. GF revised the manuscript for important intellectual content. AM has been involved in drafting the manuscript. AZS performed histological examination of the specimens. SR has given final approval of the version to be published.
